# Supplementary material for: The Japanese version of the Fear of COVID-19 scale: Reliability, validity, and relation to coping behavior
Source: PLoS One. 2020 Nov 5;15(11):e0241958. doi: 10.1371/journal.pone.0241958 (PMC7644080; doi:10.1371/journal.pone.0241958)
Supplement: S1 File — (DOCX) [file pone.0241958.s001.docx]

Japanese version of the Fear of COVID-19 Scale

以下の項目について，“全くそう思わない”，“そう思わない”，“どちらともいえない”，“そう思う”，“強くそう思う”のうち，あてはまる数字に○をつけてください。
