# Supplementary material for: The Japanese version of the Fear of COVID-19 scale: Reliability, validity, and relation to coping behavior
Source: PLoS One. 2020 Nov 5;15(11):e0241958. doi: 10.1371/journal.pone.0241958 (PMC7644080; doi:10.1371/journal.pone.0241958)
Supplement: S2 File — (DOCX) [file pone.0241958.s002.docx]

新型コロナウイルス対処行動尺度(Coping behavior)

それぞれの項目について、あなたはそれらの行動をどのくらいの頻度で行いましたか。1(まったくなかった)～6(かなりあった)のうち、当てはまる数字にそれぞれ〇をつけてください。

|  | まったくなかった | なかった | あまりなかった | 少しあった | あった | かなりあった |
| --- | --- | --- | --- | --- | --- | --- |
| (1) 換気が悪い場所には行かないようにした | 1 | 2 | 3 | 4 | 5 | 6 |
| (2) 人がたくさん集まっている場所には行かないようにした | 1 | 2 | 3 | 4 | 5 | 6 |
| (3) 他の人と、近い距離での会話や発声をしないようにした | 1 | 2 | 3 | 4 | 5 | 6 |
| (4) 手洗い・うがいやアルコールによる手や指を消毒した | 1 | 2 | 3 | 4 | 5 | 6 |
| (5) せきやくしゃみをする時は、マスク・ハンカチなどを口にあてた | 1 | 2 | 3 | 4 | 5 | 6 |
| (6) 仕事はテレワークにした | 1 | 2 | 3 | 4 | 5 | 6 |
| (7) 日常生活品をいつもより多めに買いに行った | 1 | 2 | 3 | 4 | 5 | 6 |
| (8) 食糧をいつもより多めに買いに行った | 1 | 2 | 3 | 4 | 5 | 6 |
| (9) 体温測定など自分の体調の変化を観察した | 1 | 2 | 3 | 4 | 5 | 6 |
| (10) 健康管理に気を配った | 1 | 2 | 3 | 4 | 5 | 6 |
| (11) 自分が新型コロナウイルスに感染しているかどうか、検査を受けに行った | 1 | 2 | 3 | 4 | 5 | 6 |
| (12) 体調不良になったとしても、通院を控えた | 1 | 2 | 3 | 4 | 5 | 6 |
| (13) 気を紛らわすようなことをした | 1 | 2 | 3 | 4 | 5 | 6 |
| (14) 仕事や勉強など、自分のやるべきことに集中した | 1 | 2 | 3 | 4 | 5 | 6 |
| (15) 家でできる自分の好きなことをした | 1 | 2 | 3 | 4 | 5 | 6 |
| (16) 外食を控えるようにした | 1 | 2 | 3 | 4 | 5 | 6 |
| (17) SNSやインターネットを通しての他者との関わりを求めた | 1 | 2 | 3 | 4 | 5 | 6 |
| (18) 情報を自分から遮断した | 1 | 2 | 3 | 4 | 5 | 6 |
| (19) 情報を自分から集めるようにした | 1 | 2 | 3 | 4 | 5 | 6 |

新型コロナウイルスに対する対処行動の理由 (Reasons for behaviors)

上記のような行動をとった理由についてお聞きします。それぞれの項目について、①まったくあてはまらない～⑥非常にあてはまるのうち、あてはまるもの一つに〇をつけてください。

|  | まったくあてはまらない | あてはまらない | あまりあてはまらない | 少しあてはまる | あてはまる | 非常にあてはまる |
| --- | --- | --- | --- | --- | --- | --- |
| (1) 自分が必要だと感じて行動していた | 1 | 2 | 3 | 4 | 5 | 6 |
| (2) 本当は必要ないと思いつつ、行動していた | 1 | 2 | 3 | 4 | 5 | 6 |
| (3) 自分の判断で、行動していた | 1 | 2 | 3 | 4 | 5 | 6 |
| (4) 周囲に合わせて行動していた | 1 | 2 | 3 | 4 | 5 | 6 |
| (5) ほかの人たちから言われて、そのように行動していた | 1 | 2 | 3 | 4 | 5 | 6 |
| (6) ほかの人たちからの非難をおそれて、そのように行動していた | 1 | 2 | 3 | 4 | 5 | 6 |
| (7) それらの行動をすることで、安心することができた | 1 | 2 | 3 | 4 | 5 | 6 |
