# Supplementary material for: The Japanese version of the Fear of COVID-19 scale: Reliability, validity, and relation to coping behavior
Source: PLoS One. 2020 Nov 5;15(11):e0241958. doi: 10.1371/journal.pone.0241958 (PMC7644080; doi:10.1371/journal.pone.0241958)
Supplement: Attachment — Submitted filename: Response to Reviwers.docx [file pone.0241958.s004.docx]

Octorber 1, 2020

*PLoS One*

Dear Editor:

Thank you for allowing us to submit the revised draft of our manuscript titled, “The Japanese version of the Fear of COVID-19 scale: Reliability, validity, and relation to coping behavior” to *PLoS One*. We also appreciate the time and effort you and each reviewer have dedicated to providing insightful feedback on ways to strengthen our manuscript. It is our great pleasure to resubmit our article for additional consideration. We have incorporated changes reflecting the detailed suggestions you have graciously provided. We also hope that our edits and the responses that we have provided below satisfactorily address all the issues and concerns you and the reviewers have raised.

The authors received no specific funding for this work.

To facilitate your review of our revisions, the following is a point-by-point response to the questions and comments. All changes to the manuscript are indicated in the text by yellow highlight.

Editor :

Comment 1 :

1. Please ensure that your manuscript meets PLOS ONE's style requirements, including those for file naming.

**Response**:

We have reviewed the style requirements, including the filenames, and revised the manuscript according to them.

Comment 2 :

2. Since one of the key outcomes of your study was the development of a translated version of the FCV-19S, you may wish to include this in the title of your manuscript.

**Response**:

We agree with your opinion. We have changed the title from “Variables related to fear of novel coronavirus infection (COVID-19) and coping behavior” to “The Japanese version of the Fear of COVID-19 scale: Reliability, validity, and relation to coping behavior”.

Comment 3 :

3. PLOS ONE has specific requirements for studies that are presenting a new method or tool as the primary focus, including a newly developed or modified questionnaire or scale. One requirement is that the questionnaire or scale must be openly available under a license no more restrictive than CC BY. In light of this, before we proceed, please include a copy of your questionnaire or scale as a Supporting Information file (in the original language) or provide a link if it is available through an online repository.

**Response**:

We will attach the scale we have created as a Supporting Information file (S1 File, S2 File, S1 Data).

Comment 4 :

Financial disclosure

**Response**:

We are sorry for the ambiguous explanation related to financial disclosure. Because this research was not funded, I have included the following in the cover letter. "The authors received no specific funding for this work."

Comment 5 :

PLOS requires an ORCID iD for the corresponding author in Editorial Manager on papers submitted after December 6th, 2016. Please ensure that you have an ORCID iD and that it is validated in Editorial Manager.

**Response**:

The corresponding author has created an ORCID id. I have registered it on the Editorial Manager.

Comment 6 :

6. Please include captions for your Supporting Information files at the end of your manuscript, and update any in-text citations to match accordingly.

**Response**:

We have included a caption for the Supporting Information file at the end of the manuscript and have updated the citations in the text accordingly.

Reviewer #1 :

Comment 1 : Title

I would add Japanese or japan in the title.

**Response**:

We agree with your opinion. We have changed the title from “Variables related to fear of novel coronavirus infection (COVID-19) and coping behavior” to “The Japanese version of the Fear of COVID-19 scale: Reliability, validity, and relation to coping behavior”.

Comment 2 : Abstract

Sampling procedure is missing. Real results and statistics are not reported the abstract. I cannot understand what you really mean on “were relatively comparable to those of the original FCV-19S”? how did you assess coping behavior? Please clearly report mediation results rather than writing a vague interpretation.

**Response**:

We agree with the reviewer’s point. We have made the following modifications.

(1) We have added “450 Japanese participants were recruited from a crowdsourcing platform” to the *Abstract* as the sampling procedure (p.2 lines 21-22).

(2) We have added real results and statistics to the *Abstract* (p.2 lines 28–33).

(3) We have changed the sentence from “were relatively comparable to those of the original FCV-19S” to “These results suggest that the Japanese FCV-19S is a psychometric scale with the same reliability and validity as the original FCV-19S.”

Comment 3 : Introduction

Introduction is good but needs to improving by adding some information of the latest statistics on covid-19 in japan. Please also add the following references:

**Response**:

To page 3 of the revised manuscript, we have included the latest statistics on COVID-19 from the entire world and from Japan. We have added references to lines 53–54 (Lin, 2020), lines 56–57 (Pakpour and Griffiths, 2020), and lines 62–63 (Pakpour et al., 2020).

Comment 4 :

I think that the authors need to compare their results with previous validation version.

**Response**:

Thank you for introducing us to the many references. We have cited nine references to support the validation lines 60–73. These are the following: Sakib et al. (2020), Bitan et al. (2020), Soraci et al. (2020), Winter et al. (2020), Reznik et al. (2020), Alyami et al. (2020), Haktanir et al. (2020), Nguyen et al. (2020), and Harper et al. (2020).

Reviewer #2 :

Comment 1 : Introduction

The introduction is not convincing. It fails to show the current literature gap. The aim of the study is not clear. The study should clearly specify the mentioned issues; whether you are validating FCV-19S or developing a model of COVID-19 fear effects…?

**Response**:

The reviewer has commented on the lack of persuasiveness of the *Introduction* and the lack of clarity of purpose. We agree with the reviewer’s points. We have particularly cited the references introduced by Reviewer 1 (lines 60–73) and have described the purpose as follows: *The purpose of this study was twofold. First, this study translates FCV-19S established by Ahorsu et al. [10] into Japanese and assesses reliability and validity in Japan based on a procedure equivalent to that used by Ahorsu et al. [10]. Secondly, this study develops a model of the effects of COVID-19 fear on coping behaviors, including the reasons for the behavior* (p. 5 lines 82–85).

Comment 2 : Method

How did you invite the participants to the study? How many were approached? What were the main reasons for non-participation? The rational for study sample size should be stated.

**Response**:

The reviewer has commented on the lack of clarity related to data collection in the *Materials and Methods* section. We have specifically added information related to how we recruit participants and the reasonableness of our sample size (Page 5, lines 89–101). Because we used crowdsourcing and decided to end the study when the number of participants reached 450, we cannot state the reasons for non-participants.

Comment 3 :

The rationale behind selecting the variables included in the questionnaire should be stated.

**Response**:

We agree with the reviewer’s point. We have stated the rationale for our choice of variables by citing earlier studies (Page 6, lines 118–120; Page 7, lines 132–134).

Comment 4 :

The statistical analysis should be clearly explained. The conceptual model that guided the authors for analysis should be explained in details.

**Response**:

Thank you for your comment. We realized that our original explanation was unclear and revised as follows:

(1) The *Data analysis* section described all the analyses used for this study (lines 201–230).

(2) Regarding the conceptual model, we discussed the need to measure the reasons for behavior in the *Introduction* section and the relation between FCV-19S, coping behavior, and reasons for behavior, as assumed in this study (lines 74–81). In addition, the conceptual model was explained in the *Data analysis* section (lines 222–230).

Comment 5 :

Table 1. please indicate which test was used for analysis.

**Response**:

We have provided a brief description of the analysis of the Table 1 footnote of revised manuscript. *Note: ** p<0.01, sex, smoking habit, diseases being treated, living with family and presence of persons infected were subjected to t-tests, and analysis of variance was performed for age, health condition, work status, and sources of information.*

Comment 6 :

Table 2: writing error, heart races!

**Response**:

Thank you for pointing out this error. An earlier manuscript did not present all sentences in Item 7. We have therefore added all sentences to item 7. The original FCV-19S (Ahorsu et al., 2020) also uses the word "heart races".

Comment 7 :

Why did you select model 4 as the final model? Did you change any item in the scale? What modification/s was/were made in to the scale?

**Response**:

The reviewer has commented on the lack of clarity related to the final model decision. We decided to use Model 4 as the final model because it was an acceptable fit over the other two improved models (Model 2 and Model 3) (lines 242–243). We have added a description of MI to the *Data analysis* section to show that we have assumed error correlation related to MI (lines 213–215). Some other studies of FCV-19S have also assumed error correlations (Alyami et al., 2020; Haktanir et al., 2020) (lines 335–337). We did not change the items in the scale.

Comment 8 :

Why did you use HAD for structural validity among a community population?

**Response**:

HAD has been used in the validation of FCV-19S and has been found to be reliable and valid in studies of community samples. We have added the following explanation to lines 166–168 of the revised manuscript. *Although the HADS [35] was designed originally for patients in nonpsychiatric hospital clinics, it has been found to be reliable and valid in general samples [34]. HADS was positively correlated with FCV-19S [10].*

Comment 9 :

What do you mean by (α= .93/ ω= .93) in table 3? These should be explained.

**Response**:

We have provided a brief description of α and ω in the Table 2 and Table 3 footnotes. We have also given the use of α and ω as indicators of reliability in *Data analysis* section (lines 215–216).

Comment 10 :

The main concern is that, could we use the same sample for both validation study and SEM?

**Response**:

The reviewer has asked about the propriety of using the same sample for validation and SEM.

Many other studies have applied factor analysis and other analyses (including SEM) to the same samples. Therefore we believe there is no particular problem with their application here.

Comment 11 :

What are the differences between table 4 and 5?

**Response**:

Table 4 presents results of exploratory factor analysis for “coping behavior.” Table 5 shows the results of exploratory factor analysis for “reason for behavior.” For “reason for behavior,” we changed the factor names to clarify it that it is a reason (Previous manuscript: self-determination and conformity behavior. Revised manuscript: self-determining reason and conformity reason).

Comment 12 :

The discussion is highly poor. In this part, you should compare the results obtained from your study with similar studies either confirming or rejecting the current results. Then, express your explanations or justifications.

**Response**:

The reviewer is concerned about the lack of sufficient discussion. The reviewer is correct. We appreciate the chance to clarify our exposition. We have revised the paper as explained below.

(1) Results of the confirmatory factor analysis are discussed in comparison to results obtained for other countries (lines 335–346).

(2) The relation between sociodemographic variables and FCV-19S was discussed in comparison to the results of earlier studies (lines 349–358).

(3) Effects of FCV-19S on coping behaviors were discussed in comparison to Winter et al., 2020 and to Harper et al., 2020 (lines 359–375 ).

**Other points to change**

ERROR CORRECTED

p.6 line 103: “Socio-demographic variable” (previous manuscript: Attributes of the respondents)

p.6 line 108: “6 = having other symptoms” (previous manuscript: 9 = having other symptoms)

p.11 line 236: “FCV-19S-J” (previous manuscript: Japanese FCV-19S-J)

p.23 line 389: “longitudinal study” (previous manuscript: cross-sectional surveys)

Again, we thank you for giving us the opportunity to strengthen our manuscript with your valuable comments and queries. We have worked diligently to incorporate your feedback, and hope that these revisions will facilitate the acceptance of our submission.

Sincerely,

Koubun Wakashima, Ph.D.

Tohoku University Graduate School of Education

27-1 Kawauchi, Aoba-ku, Sendai, Miyagi 980-8576, Japan

Phone/Fax: +81-(0)22-795-6139

E-mail: kobun.wakashima.d3@tohoku.ac.jp
